# Data on insect biodiversity in a Chinese potato agroecosystem from DNA metabarcoding

**DOI:** 10.1038/s41597-025-04452-8

**Published:** 2025-01-22

**Authors:** Changjin Lin, Chenxi Liu, Lilin Chen, Hongmei Cheng, Muhammad Ashfaq, Paul D. N. Hebert, Yulin Gao

**Affiliations:** 1 Sino-American Biological Control Laboratory, Institute of Plant Protection, Chinese Academy of Agricultural Sciences, Beijing, 100193 PR China; 2 College of Life Sciences, Fujian Agriculture and Forestry University, Fuzhou, Fujian 350002 PR China; 3 State Key Laboratory for Ecological Pest Control of Fujian and Taiwan Crops, Institute of Applied Ecology, Fujian Agriculture and Forestry University, Fuzhou, Fujian 350002 PR China; 4 Centre for Biodiversity Genomics and Department of Integrative Biology, University of Guelph, Guelph, ON N1G 2W1 Canada; 5 State Key Laboratory for Biology of Plant Disease and Insect Pests, Institute of Plant Protection, Chinese Academy of Agricultural Sciences, Beijing, 100193 PR China

**Keywords:** Biodiversity, Population dynamics

## Abstract

Potato (*Solanum tuberosum*) is a staple crop important in global food security. As a leading potato producer, China faces significant challenges from insect pest infestations that compromise yield and quality. However, insect communities within Chinese potato fields remain poorly characterized. This study aimed to explore insect diversity in potato fields in Yunnan Province. From autumn 2021 to summer 2022, five Malaise traps were strategically deployed to capture insect samples. In total, 245 samples were collected over 49 weeks, and DNA metabarcoding was performed on bulk samples. The generated sequences were curated and analyzed using the Barcode of Life Data System and the Multiplex Barcode Research and Visualization Environment. The analysis assigned sequences to 1,688 Barcode Index Numbers (BINs) as species proxies derived from the Global Insecta Library, along with 166 BINs from the China Insecta dataset. This research provides valuable insights for barcoding local biodiversity and developing regional reference libraries and presents a comprehensive dataset of insect biodiversity within potato agroecosystems, encompassing 1,707 BINs linked to known insect taxa.

## Background & Summary

*Solanum tuberosum* L., commonly known as the potato, is an annual herbaceous plant belonging to the Solanaceae family. It originates from the Andes Mountains in South America^1,2^. Potatoes rank as the fourth largest food crop globally, following wheat, rice, and corn, and play a crucial role in global food security^3,4^. Cultivated in approximately 160 countries or regions worldwide, potatoes serve as vegetables in developed nations and as staple foods in developing countries^4,5^. Notably, China possesses one of the largest potato cultivation areas, contributing 25.1% to global production and 31.9% to total global planting area^6,7^.

The expansion of potato cultivation in China has intensified the prevalence of potato pests. Annually, about 2.6 million hectares are affected, representing 47.3% of the potato planting area, leading to an average annual yield loss of 196,000 tons^8^. The primary pests include *Phthorimaea operculella*^9,10^, *Leptinotarsa decemlineata*^11^, *Henosepilachna vigintioctopunctata*^12^, and wireworms^13^. However, data on insect communities generated from comprehensive and systematic surveys in potato agroecosystem remain lacking, significantly hindering progress on pest monitoring and management. In addition, most of the current studies have focused on pest insects^14–18^, often neglecting natural enemy insects which play vital roles in pest biological control and serve as an essential indicator for assessing agricultural biodiversity and ecosystem service function^19,20^. Understanding the presence of natural enemy insects in potato field ecosystems remains largely unexplored. A previous study identified Barcode Index Numbers (BINs) linked to known species of Diptera and Hymenoptera, which may have potential as biological control agents^21^. Therefore, bridging this gap by coupling metabarcoding with the BIN system to explore natural enemies in potato agroecosystem is a feasible approach. Recognizing the diversity and occurrence dynamics of pests and beneficial insects in potato agroecosystem, monitoring pests or invasive species, and formulating measures for the protection of green production are pivotal for ensuring food security and promoting sustainable agricultural practices.

Traditionally, biodiversity identification has relied on visual observations, morphological species identification, and individual counting. However, these methods can be hampered by insufficient morphological identification resources, taxonomic expertise, and standardized sampling techniques^22–24^. DNA barcoding^25^, a technique utilizing conserved DNA fragments for species identification, has revolutionized biodiversity studies by enhancing the speed, accuracy, and cost-effectiveness of specimen classification. DNA barcoding, along with DNA metabarcoding—an approach involving genetic material extraction from mixed samples, amplification, sequencing, and comprehensive analysis via high-throughput sequencing (HTS)—has been instrumental in characterizing terrestrial and aquatic insect communities^26–30^. The efficacy of DNA barcoding for species identification depends on comparisons with DNA reference libraries to ascertain species information^31,32^. The Barcode of Life Data System (BOLD; https://www.boldsystems.org/) is a global public repository for DNA barcodes. The system assigns barcode sequences meeting specific criteria (>507 bp, <1% fuzzy bases, no stop codons, no contamination) to BINs, serving as species proxies^33^. The implementation of the BIN system has expedited species recognition and counting via DNA barcoding^34–36^.

For DNA metabarcoding, bioinformatics typically involves a pipeline that converts HTS data into an OTU table for downstream analysis. Tools for DNA metabarcoding analysis, such as SLIM (https://trtcrd.github.io/SLIM/), QIIME (https://docs.qiime2.org/2024.5/), Galaxy (https://usegalaxy.org/), FROGS (https://frogs.toulouse.inra.fr/), and Multi Barcode Research and Visualization Environment (mBRAVE; https://mbrave.net/) have been widely used in insect biodiversity studies. However, each pipeline has its own specific philosophy, strengths, and limitations, which should be considered based on the aims of a specific study, as well as the bioinformatics expertise of the user. Owing to a module-centered organization, SLIM can be used for a wide range of metabarcoding cases and can also be extended by developers for custom needs or for the integration of new software^37^. QIIME2, initially developed to analyze microbiome data, is a toolbox which is dependent on plugins used^38^. Galaxy is an open source, web-based platform designed for data intensive biomedical research, providing a graphical user interface for specifying what data to operate on, what steps to take, and what order to do them in^39^. The user-friendly and Galaxy-supported pipeline FROGS can analyze large sets of DNA amplicons sequences accurately and rapidly, essential for biodiversity^40^.The mBRAVE is a cloud-based platform supporting the storage, validation, analysis, and publication of highly multiplexed projects based on HTS instruments. This platform allows parallel sequence identification for the cytochrome c oxidase 1 (COI) barcoding region by simultaneous comparison to the BOLD for millions of sequences^41^. As the BIN system has offered a novel approach to circumvent morphological bottlenecks to discriminate species, pairing of BINs with HTS in mBRAVE platform was widely used on biodiversity assessments.

**Fig. 1:**
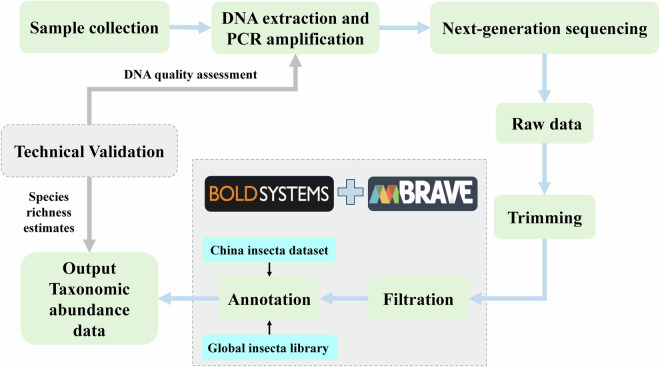
Overview of data generation from sample collection to the validation of data generation.

From August 2021 to July 2022, spanning two potato growing seasons with two fallow periods, we utilized a standardized DNA barcode method to process and analyze potato field insects collected in Yunnan Province, China (Fig. 1). This study aimed to elucidate the insect community structure in potato fields at different growth stages and the occurrence dynamics of major pests and natural enemies, providing data support for beneficial insect resource development and pest monitoring and management. The core research dataset maps each sample to a BIN, comprising 1707 BINs related to known insects. While most BINs were assigned to a single taxon, 22.5% were linked to more than one species or genus. The lower number of BINs with species matches on BOLD indicates the incompleteness of the current barcode reference library, emphasizing the need to develop regional DNA barcode reference libraries. Meanwhile, comparative analysis with the China Insecta dataset and the Global Insecta Library underscores the importance of barcoding local biodiversities and developing regional libraries. Moreover, the dataset also included insect BINs recovered from autumn and spring fallow periods after potato harvests, which can highlight the importance of insect conservation and is used to explore the ecology on population occurrence and succession. In addition, the present study only examined one region, which is a representative area in southern China. Constructing a comprehensive insect inventory for potato agroecosystem across China will require surveys at the representative sites in other potato areas. Our findings offer detailed insights into insect biodiversity in Chinese potato fields for the first time, furnishing valuable data for managing potato pests and assessing beneficial insects.

## Methods

### Sample collection

**Fig. 2:**
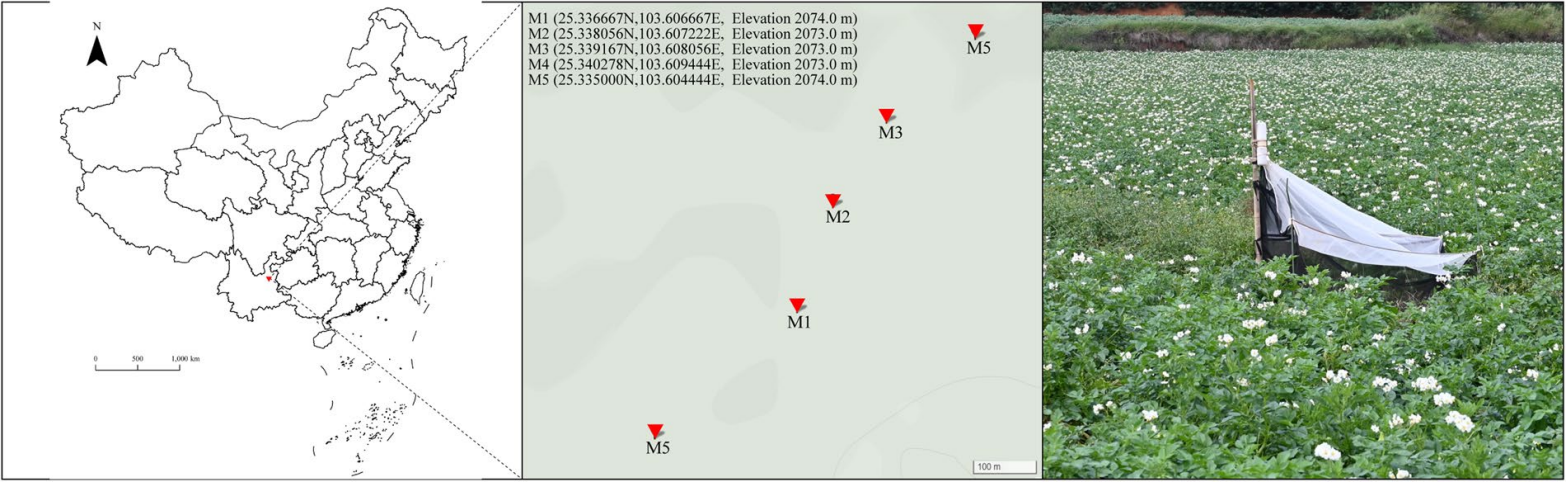
Dataset displaying the sampling points of insects in the potato field of Qvjing City, Yunnan Province, China.

From August 5, 2021, to July 14, 2022, five Malaise traps (M1, M2, M3, M4, and M5) were deployed in potato fields in the Malong District, Qvjing City, Yunnan Province, China (Fig. 2). Insect samples were primarily collected using bottles attached to the Malaise traps. These traps are designed to guide insects into the collection bottles, which contained 250 mL of 95% ethanol to preserve the specimens. The collection bottles were replaced weekly and each replacement was documented. For example, samples collected in the first week at sampling point M1 were labeled as “M1B1,” while those collected in the second week were labeled “M1B2,” and so forth. A total of 245 samples were collected over the 49-week sampling period. Upon returning to the laboratory, the ethanol in the collection bottles was replaced to ensure optimal preservation of the samples for subsequent molecular analyses.

### DNA barcode sequencing

We used the 5′ region of cytochrome c oxidase 1 (COI) mitochondrial marker, which is the standard barcoding marker for animals (BOLD, http://www.boldsystems.org). Total genomic DNA was extracted from samples with limited tissue using the TIANamp Micro DNA Kit (Tiangen Biotech Co., Ltd., Beijing, China). For samples with abundant tissue, we employed a membrane-based protocol modified for bulk samples^28,42^.

The DNA extraction method involved the following steps:

1. The bulk insect sample was placed in a 100 mL centrifuge tube, dried of residual alcohol, mixed with an appropriate amount of lysis solution, and incubated in a constant-temperature shaker at 56 °C and 120 r/min for 36 h.
2. After lysis, 150 μL of the insect lysis solution was mixed with 300 μL of binding mix (BM) in a sterile centrifuge tube, shaken well, and transferred to an adsorption column. This process was repeated in triplicate for each sample. The mixture was centrifuged at 5000 *g* for 2 min.
3. The sample was washed with 300 μL of Protein Wash Buffer (PWB) and centrifuged at 5000 *g* for 2 min.
4. The adsorption column was washed with 600 μL of Wash Buffer (WB) and centrifuged at 5000 *g* for 4 min.
5. Step 4 was repeated.
6. The adsorption column was transferred to a clean centrifuge tube and centrifuged at 10000 *g* for 4 min to remove any residual buffer. It was then transferred to a clean collection tube and incubated at 56 °C for 30 min to dry the membrane.
7. DNA was eluted by adding 50 μL of 10 mM Tris HCl (pH 8.0) and centrifuged at 10000 *g* for 5 min.
8. The adsorption column was discarded, and DNA was quantified using a microspectrophotometer (SH-NanoOne). Qualified DNA samples were stored at −20 °C for future use.

During the extraction process, DNA from each sample was extracted by taking three subsamples (denoted as X1, X2, and X3), resulting in a total of 726 samples for sequencing. A fragment of the COI barcode was amplified using degenerate primers BF3 (CCHGAYATRGCHTTYCCHCG) and BR2 (TCDGGRTGNCCRAARAAYCA)^43^. Sequencing was performed by Personal Biotechnology Co., Ltd. (Shanghai, China), using the Illumina NovaSeq machine with the NovaSeq6000 SP Reagent Kit (500 cycles) for 2 × 250 bp paired-end sequencing.

### Bioinformatics analysis pipeline

After obtaining the raw data, Cutadapt (v2.3) was used to remove primer fragments from the sequences, setting the range from 0 to 10. Sequences with mismatched primers were discarded. The mBRAVE^41^ platform was used to analyze the metabarcoding data as following steps:

1. A new project was created by introducing the title and a small description of the project
2. Uploaded sequences were controlled and filtered by setting analysis parameters.
  2.1 Trimming (only trimming by length was applied):
  Trim front: 0 bp
  Trim end: 0 bp
  Primer masking: off
  2.2 Filtering (removal of low-quality reads for an average quality value – QV - less than 20 or sequences shorter than 200 bp):
  Min QV: 20 qv
  Min Length: 200 bp
  Max Length (Pre-Trim):1000 bp
  Max Bases with Low QV (<20):25%
  Max Bases with Ultra Low QV (<10):5%
  2.3 Other parameters (after removal of low-quality reads and chimeras, the reads were clustered into BINs):
  Dereplication Min Rep:1
  Pre-Clustering Threshold: None
  ID Distance Threshold: 3%
  ID Min Overlap:100 bp
  Exclude From OTU Threshold:3%
  Minimum OTU Size:3OTU Threshold:2%
  Read Sub-Sampling - Max Reads per Sample:5000
  Read Sub-Sampling - Max Reads per Contig:500
  Paired End (optional - Illumina instruments only)
  Paired End Merging: Merge
  Assembler Min Overlap: 20 bp
  Assembler Max Substitution:5 bp
3. Global Insecta Library (SYS-CRLINSECTA) and the China Insecta dataset (DS-CHINAINS) as the reference database were added. For annotation, the built-in reference databases on the mBRAVE platform: the Global Insecta Library (SYS-CRLINSECTA) and the China Insecta dataset (DS-CHINAINS) were selected. The Global Insecta Library contains 1,236,890 sequences, 864,126 BINs, and 249,880 insect species. The China Insecta dataset (DS-CHINAINS) contains 90,705 sequences, 18,337 BINs, and 9,290 species.
4. Sequence uploads
  Select the “Upload run” module, edit the Sample name and group.
  Sample type: Passive Open Trap(terrestrial)
  Run type: mixed
  Instrument: Illumina NovaSeq
5. The results of the best matches obtained with each reference library used in the analysis were downloaded for each sample. The BINs table were organized and created in an Excel file. Only taxonomic matches above 97% were considered. Reads were retained for further analysis only if ≥5 reads matched to BINs in the table.

## Data Records

The dataset, titled “Raw data for insect DNA metabarcoding sequences in potato fields in China”, were deposited in the National Center for Biotechnology Information (NCBI) Sequence Read Archive under accession number SRP538027^44^.

1. Raw fastq data: This file contains 1,452 raw sequencing fastq files, including both forward and reverse reads for each sample. The fastq data are shared to allow other researchers to validate, reprocess, and reanalyze the data based on their analysis needs and custom parameters.
2. Sample metadata: This file includes basic information about the samples in this study, such as the position information for 5 Malaise traps (latitude, longitude, altitude), collection times, and corresponding sample numbers. Due to the absence of insects in samples M1B29, M3B30, and M5B27, molecular experiments were conducted on the remaining 242 samples.

## Technical Validation

### Data reliability

First, three rounds of DNA extraction and amplification were performed for each collected sample to maximize DNA yield from all species present. Second, after completing DNA extraction, Thermo Scientific NanoDrop instrument was used to evaluate the quality and concentration of the extracted DNA. The amplification length of the COI gene fragment was verified using 1.2% agarose gel electrophoresis and visualizing bands. Finally, high-quality reads were obtained using the mBRAVE platform, where double-ended sequences were merged, and quality control and filtering were performed. The mBRAVE platform, integrated with the BOLD system, assigns sequences to corresponding BINs through a specific algorithm to generate a BIN table for each sample. Only BINs represented by five for more reads were retained.

**Fig. 3:**
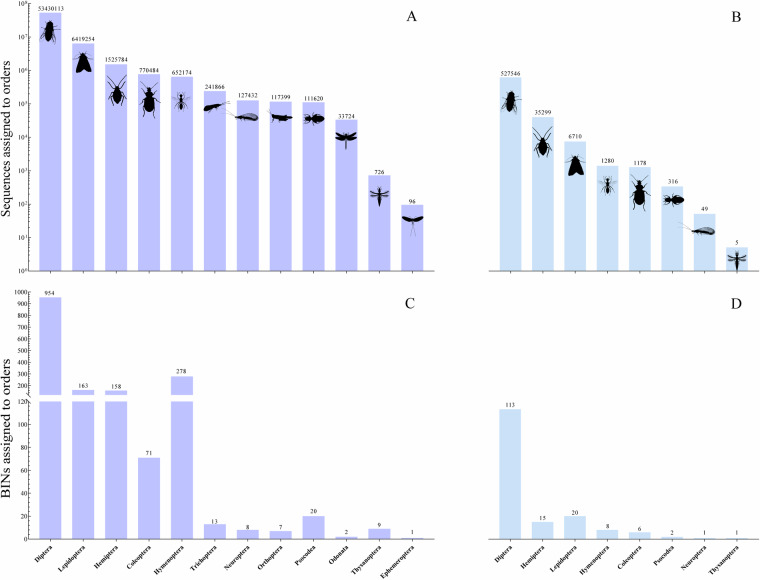
All samples were annotated with insect sequences of different orders using the mBRAVE platform. (**A**) Number of sequences in the Global Insecta Library; (**B**) Number of sequences in the China Insecta dataset; (**C**) Number of BINs in the Global Insecta Library; (**D**) Number of BINs in the China Insecta dataset.

**Fig. 4:**
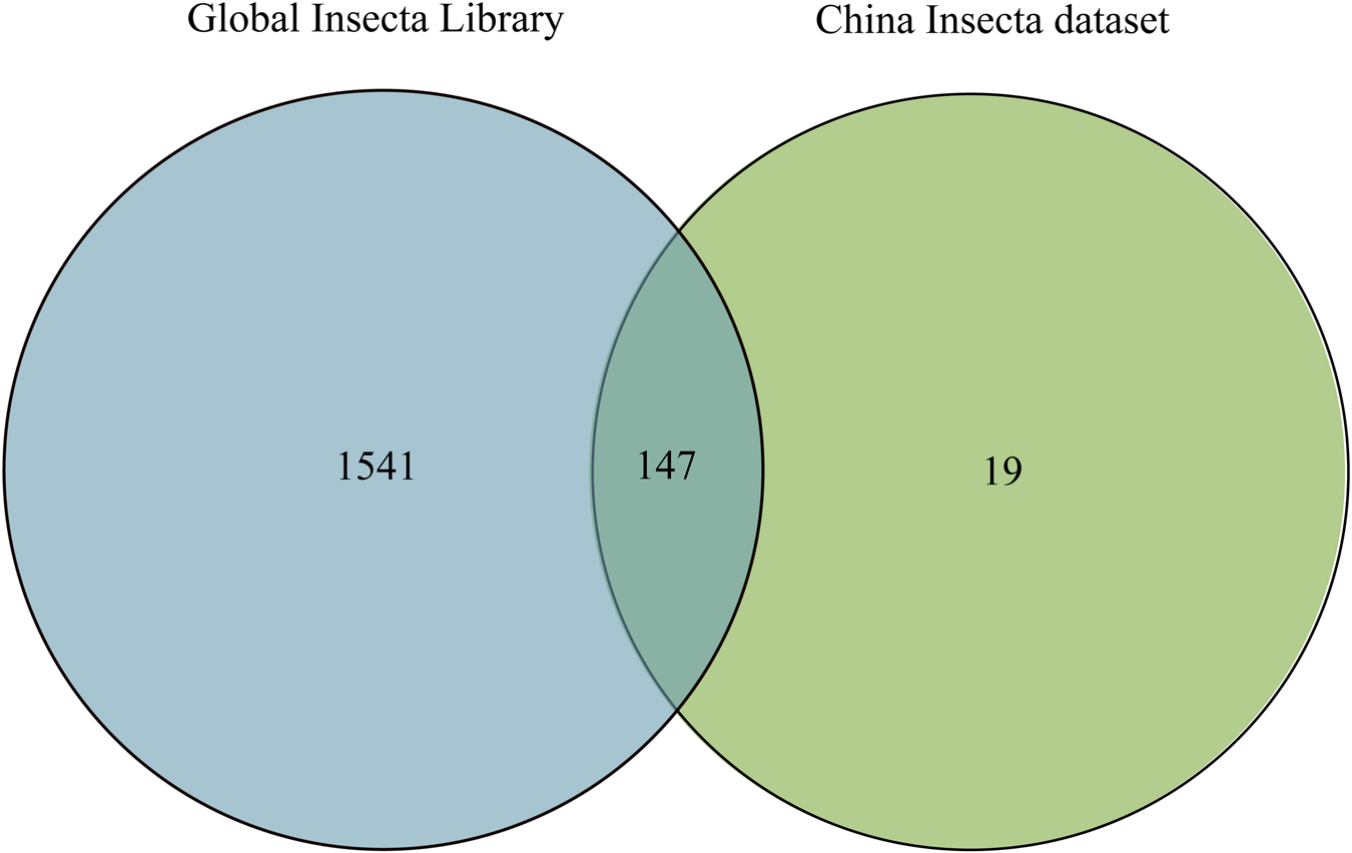
Venn diagrams depicting the overlaps in BINs belonging to China Insecta dataset and Global Insecta Library.

Figure 3A–D illustrate the number of sequences and BINs matching with the insect orders in the Global Insecta Library and China Insecta dataset, respectively. The 63,330,672 sequences from this study were matched with 1,684 BINs in the Global Insecta Library. After removing four BINs due to uncertain order-level taxonomy, these BINs were linked to 12 insect orders, with the majority (84%) belonging to Diptera. In contrast, only 572,383 sequences were matched with 166 BINs in the China Insecta dataset, covering just eight insect orders. Of these, 527,546 sequences (92%) were matched to Diptera. Figure 4 shows that the BINs identified in the Global Insecta Library almost entirely overlap with those identified in the China Insecta dataset, except for 19 BINs, highlighting the necessity of developing regional libraries.

**Fig. 5:**
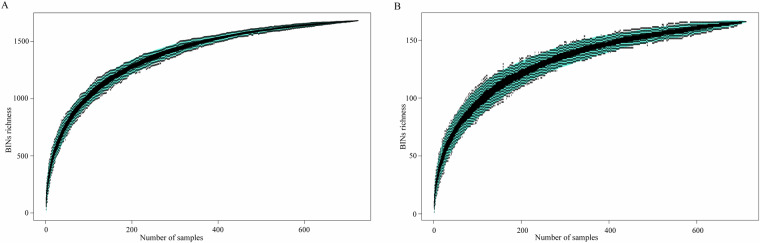
Gradually flattening cumulative curve indicating that most of the insects in the potato field had been sampled. (**A**) Number of BINs in Global Insecta Library; (**B**) Number of BINs in China Insecta dataset.

**Fig. 6:**
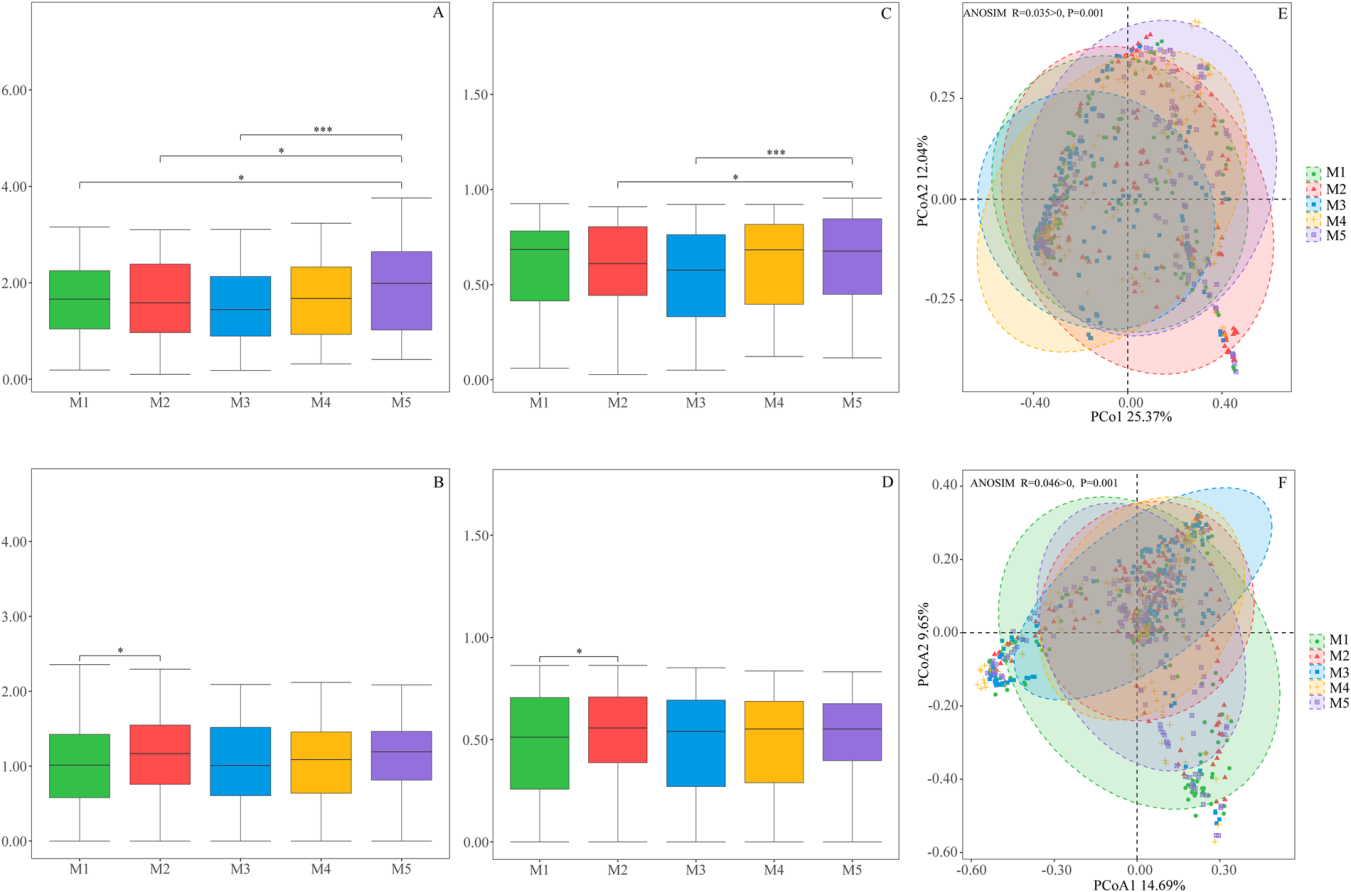
Comparisons of insect composition and differences between the Global Insecta Library and China Insecta dataset. Alpha diversity (**A**–**D**) comparisons of the diversity of insects (the Shannon and Simpson indices). Beta diversity (**E** and **F**). Principal coordinate analysis based on Bray‒Curtis distance showing patterns of separation in the insects communities at five sampling sites in the Global Insecta Library and China Insecta dataset. PC1 and PC2 represent the top two principal coordinates, and the explanation of diversity is expressed as a percentage. Each point represents a sample and is colored based on the five sampling sites.

The sampling efficiency of this study was analyzed by establishing a cumulative curve (Fig. 5A,B). The gradually flattening curve indicates that most insects in the potato field were sampled. Comparisons of insect composition and differences between the Global Insecta Library and China Insecta dataset were conducted based on the taxonomic abundance data. Differences among the five sampling sites between the Global Insecta Library and China Insecta dataset were observed in the alpha diversity analysis, using the Simpson and Shannon indices (Fig. 6A–D). Beta diversity analysis, based on the Bray-Curtis distance and the Adonis test, revealed underlying similarities in insect biodiversity across the five sampling sites when using both the Global Insecta Library and China Insecta dataset (Fig. 6E,F).

## Supplementary information

Supplementary Tables

## Data Availability

Analyses involving cumulative curve were drawn; alpha diversity indices, dissimilarity matrices, principal coordinate analysis, and Adonis were performed using the function of the R “vegan” package^45^ and the scirpts were deposited in GitHub (https://github.com/Lin-CJ-2024/Alpha-and-Beta-diversity).
