# Supplementary material for: Data on insect biodiversity in a Chinese potato agroecosystem from DNA metabarcoding
Source: Sci Data. 2025 Jan 22;12:131. doi: 10.1038/s41597-025-04452-8 (PMC11754625; doi:10.1038/s41597-025-04452-8)
Supplement: Supplementary file 1 — Supplementary Tables [file 41597_2025_4452_MOESM1_ESM.docx]

**Supplementary Information**

### Data on insect biodiversity in a Chinese potato agroecosystem from DNA metabarcoding

Changjin Lin ^1,2,3^, Chenxi Liu ^1*^, Lilin Chen ^3^, Hongmei Cheng ^1^, Muhammad Ashfaq ^4^, Paul D. N. Hebert ^4^, Yulin Gao ^5^

^1^Sino-American Biological Control Laboratory, Institute of Plant Protection, Chinese Academy of Agricultural Sciences, Beijing, PR China, 100193

^2^College of Life Sciences, Fujian Agriculture and Forestry University, Fuzhou, Fujian, PR China, 350002

^3^State Key Laboratory for Ecological Pest Control of Fujian and Taiwan Crops, Institute of Applied Ecology, Fujian Agriculture and Forestry University, Fuzhou, Fujian, PR China, 350002

^4^Centre for Biodiversity Genomics and Department of Integrative Biology, University of Guelph, Guelph, ON, Canada, N1G 2W1

^5^State Key Laboratory for Biology of Plant Disease and Insect Pests, Institute of Plant Protection, Chinese Academy of Agricultural Sciences, Beijing, PR China,100193.

*Corresponding author(s): Chenxi Liu (liuchenxi@caas.cn)

**Table S1. Sample collection metadata.**

**Table S2. The relative abundances for insect BINs**
